# Tectorial membrane: structure, function, and its implications for hearing loss

**DOI:** 10.3389/fneur.2025.1630549

**Published:** 2025-08-18

**Authors:** Panpan Bian, Jiong Dang, Bai-cheng Xu

**Affiliations:** Department of Otolaryngology-Head & Neck Surgery, The Second Hospital & Clinical Medical School, Lanzhou University, Lanzhou, Gansu, China

**Keywords:** tectorial membrane, hearing loss, cochlea, genetic mutations, ototoxicity, aging, thyroid hormone

## Abstract

The tectorial membrane (TM) is an essential extracellular matrix in the cochlea, integral to auditory processing by facilitating hair cell stimulation and sound transmission. Despite its vital role, the mechanisms underlying TM-related hearing loss remain unclear. This review aim to discuss the structure and functions of the TM, exploring its role in cochlear mechanics and auditory signal amplification. Abnormalities in TM composition, including disruptions in collagen, glycosaminoglycans, and non-collagenous proteins, are implicated in various forms of hearing loss, including those associated with genetic mutations and ototoxic drug exposure. We also examine the contributions of genes such as *TECTA*, *TECTB*, and *CEACAM16*, whose mutations disrupt TM integrity and lead to sensorineural hearing loss. Additionally, the impact of aging and thyroid hormone deficiency on TM degeneration is considered. Current diagnostic and therapeutic approaches are discussed, with an emphasis on the potential of gene therapy and stem cell therapy.

## 1 Introduction

The tectorial membrane (TM) is a critical component in the auditory system, playing a pivotal role in stimulating hair cells and facilitating sound transmission. Abnormalities in the TM are commonly associated with otological disorders, with a reported prevalence of 21.9% based on studies of human temporal bones. Conditions such as idiopathic sudden deafness (57.1%), genetic etiologies (53.7%), and ototoxicity (40.0%) have been linked to TM abnormalities, while presbycusis is relatively uncommon, accounting for only 2.9% of cases (1). Despite its recognized importance, the specific mechanisms by which TM proteins contribute to hearing loss remain underexplored.

As an extracellular matrix located above the organ of Corti, the TM is composed of water, glycosaminoglycans, collagenous fibers (primarily types II, IX, and XI), and non-collagenous proteins that form a striated-sheet matrix (SSM). This complex ultrastructure is essential for the TM’s role in sound transmission and amplification. Furthermore, its ability to regulate the ionic environment around hair-cell stereocilia underscores its critical function in auditory processing.

This review provides a comprehensive analysis of the TM, including its structural components, proteins, genetic mutations, and their roles in hearing loss, as well as current therapeutic strategies. It also addresses existing knowledge gaps and suggests potential future research directions to advance the diagnosis and treatment of TM-related auditory disorders.

## 2 Physiological and structural overview of TM

### 2.1 The composition of TM

TM is a highly hydrated extracellular matrix located above the mechanosensory hair cell bundles in the cochlea. The TM’s three main constituents are water (97%), glycosaminoglycans and collagen fibers (collagen II, IX, and XI), and non-collagenous proteins, such as *α*-tectorin, *β*-tectorin, CEACAM16, otogelin, and otogelin-like. The prominent feature of the TM is the thick collagen bundles running radially. These bundles are composed of 20 nm diameter collagen filaments imbedded in a tectorin-based striated-sheet matrix (2). The TM is closely associated with the stereocilium of the outer hair cells.

### 2.2 The function and mechanism of TM

The mechanical connection between the TM and outer hair cells (OHCs) plays a crucial role in cochlear amplification. By stimulating the OHCs through contact with their stereociliary bundles, the TM facilitates synchronized movement of the OHC stereocilia, allowing efficient mechanical energy transfer. The horizontal top connectors, formed by otogelin, otogelin-like, and stereocilin proteins, are essential for maintaining the cohesion of OHC stereocilia and stabilizing the mechanical coupling between the TM and OHCs (3). These proteins interact together to maintain the mechanical integrity of OHC stereocilia, underscoring the importance of this coupling in cochlear amplification and mechanotransduction (3, 4).

**Figure 1:**
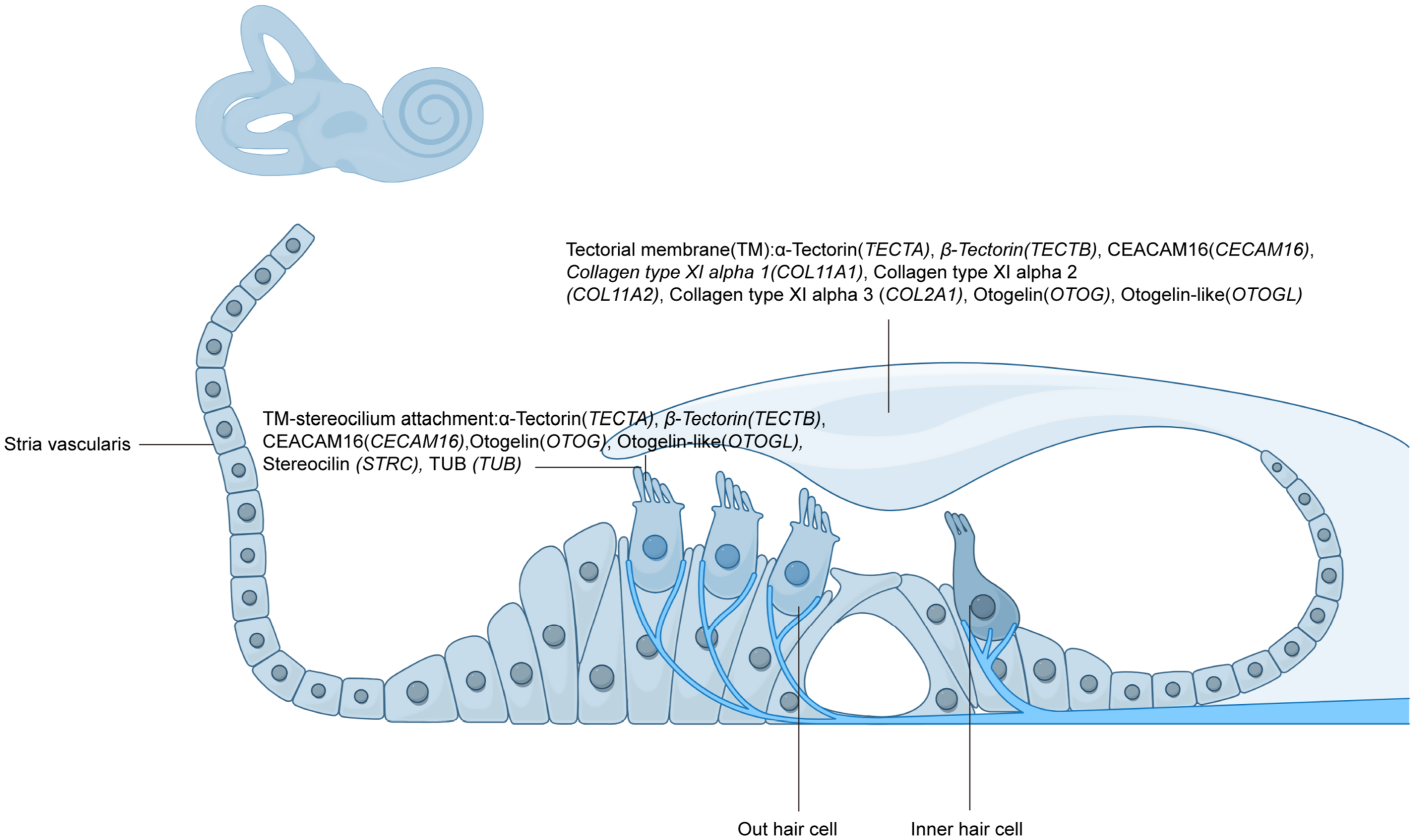
Diagram illustrating the structure of the cochlea and the attachment of the tectorial membrane (TM) to the stereocilia of hair cells. The TM is composed of various proteins, including *α*-tectorin (*TECTA*), *β*-tectorin (*TECTB*), CEACAM16 (*CECAM16*), collagen types XI alpha 1, 2, and 3 (*COL11A1, COL11A2, COL2A1*), otogelin (*OTOG*), and otogelin-like (*OTOGL*). These proteins are essential for the attachment and stability of the TM to the stereocilia, specifically of the outer hair cells (OHCs). The diagram also depicts the TM-stereocilium interaction and its role in cochlear mechanics, including the involvement of key proteins such as stereocilin (STRC) and TUB protein. Additionally, the inner hair cells (IHCs) are shown, highlighting their position in relation to the TM and the outer hair cells.

The relationship between the tectorial membrane (TM) and inner hair cells (IHCs) has traditionally been considered indirect, with the TM modulating IHC responses via endolymphatic fluid motion or through its mechanical interaction with outer hair cells (OHCs) (5). However, recent high-resolution imaging studies have fundamentally challenged this classical view. Using laser confocal reflectance and fluorescence microscopy in ex vivo guinea pig cochleae, Hakizimana and Fridberger provided compelling anatomical evidence that IHC stereocilia, like those of OHCs, are physically embedded within the TM (6). Overlay analyses of reflected and labeled images revealed consistent continuity between the TM and stereociliary bundles in both IHC and OHC regions across multiple preparations (Figure 1).

This discovery redefines the mechanical interface of IHCs, indicating that their activation may not depend solely on hydrodynamic shearing forces but also on direct radial displacements transmitted through the TM. Such direct coupling could facilitate enhanced sensitivity, phase-locked stimulation, and improved frequency selectivity, particularly in the apical cochlea. Furthermore, Ca^2+^ ratiometric imaging revealed that the TM functions as a localized calcium reservoir. Stereocilia of both IHCs and OHCs were shown to reside within microdomains of elevated Ca^2+^ concentration, despite the typically low Ca^2+^ levels of surrounding endolymph. This TM-mediated Ca^2+^ enrichment may resolve the long-standing paradox of high MET channel open probabilities under low Ca^2+^ conditions and implies that the TM supports both mechanical and ionic prerequisites for efficient mechanotransduction.

Importantly, during acoustic stimulation, IHC and OHC stereocilia exhibited synchronized phase-locked motion within the TM, despite distinct local kinematic patterns. This further supports the hypothesis that the TM plays a direct and dynamic role in transducing mechanical stimuli to IHCs. Moreover, the TM itself propagates traveling waves (6–8), suggesting that its longitudinal motion may contribute to fine-tuned spatial activation of hair cells. Collectively, these findings warrant a revision of canonical models of cochlear micromechanics, positioning the TM as a critical structural and biochemical mediator in both OHC and IHC function (Figure 1 and Table 1).

**Table 1:** Summary of tectorial membrane proteins, their functions, genetic associations, and clinical significance in hearing loss.

Protein	Function	Gene	Clinical significance				References
			Related hearing disease	Inheritance	Type of NSHI	Audiological phenotypes	
α-tectorin	It is one of the main non-collagenous components of the TM; It is necessary for the formation, coalignment and orientation of the first collagen-fibril bundles	*TECTA*	DFNA8/12; DFNB21; Jacobsen syndrome; Family Meniere disease	AD or AR	prelingual or postlingual	moderate to profound; mid- or high- or all frequencies	Yasukawa et al. (17)Choi et al. (18)Fryns et al. (19)Kim et al. (20)Boucher et al. (21)
β-tectorin	It is one of the main non-collagenous components of the TM and It may be affect the motion of the TM, especially radial motion	*TECTB*	It is a candidate gene for hearing loss	—	—	—	Xu et al. (22)Cheatham et al. (23)
CEACAM16	It is one of the main non-collagenous proteins of the TM; It interact in complicated ways to ensure proper hair cell activation and to stabilize the active process with α-tectorin and β-tectorin; It is correlative with age-related degeneration of the tectorial membrane	*CEACAM16*	DFNA4B; DFNB113	AD or AR	postlingual	mild to profound progressive; mid- or high- or all frequencies	Cheatham et al. (24)Markova et al. (25)Goodyear et al. (26)
otogelin	It is one of non-collagenous components of the TM; It leads that outer hair cell bundles are anchored in the attachment crowns of the TM; It works in the early development of the TM	*OTOG*	DFNB18B; Noise-induced hearing loss; Familial Meniere’s disease	AR	prelingual	moderate to severe, mid frequency	Avan et al. (3), Schraders et al. (37), Zhang et al. (38)
otogelin-like	It is one of non-collagenous components of the TM; It leads that outer hair cell bundles are anchored in the attachment crowns of the TM;work in the later development of the TM	*OTOGL*	DFNB84B; Vertigo	AR	prelingual	moderate, mid- to high-frequency	Avan et al. (3). Yariz et al. (35)
otoancorin	It represents a group of non-collagenous glycoproteins of the TM; It is required for adhesion of the TM to the spiral limbus; It may be associated with attachment of the IHC stereocilia to the TM	*OTOA*	DFNB22	AR	prelingual	moderate to profound; all frequencies	Zwaenepoel et al. (42), Hakizimana and Fridberger (6)
collagen type XI alpha 1	It forms radial collagen fibrils of the TM	*COL11A1*	DFNA37; MRSHS; Stickler syndrome type II	AD or AR	prelingual or postlingual	progressive; mild to moderate; mid- to all frequencies	Booth et al. (29)Richards et al. (31)
collagen type XI alpha 2	It forms radial collagen fibrils of the TM	*COL11A2*	DFNA13, DFNB53; Stickler syndrome type III	AD or AR	prelingual	non-progressive or progressive; mid- to all frequencies	Ala-Kokko et al. (30)
collagen type XI alpha 3	It forms radial collagen fibrils of the TM	*COL2A1*	Stickler syndrome type I; Stickler syndrome type I nonsyndromic ocular	AD	postlingual	mild; high-frequency	Ala-Kokko et al. (30)
stereocilin	It is essential to the formation of horizontal top connectors	*STRC*	DFNB16; DIS	AR	Early onset	mild to moderate, mid- to high-frequency, progressive	Vona et al. (46)
TUB protein	It maintains *STRC* localization at the tips of stereocilia as well as the integrity of STRC-dependent stereociliary links such as horizontal top connectors and TM- attachment crowns	*TUB*	It is a candidate gene for hearing loss	—	—	—	Han et al. (48)

### 2.3 The role of the tectorial membrane in Cochlear micromechanics and hearing loss

TM plays a pivotal role in cochlear micromechanics, which is essential for sound amplification and the efficient transmission of auditory signals (9). Radial motion within the organ of Corti is tuned to the frequency of cochlear amplification rather than the frequency of the underlying basilar membrane. Radial tuning of the reticular lamina (RL) and TM has been observed even in non-functional cochleae, such as in dead cochleae and Tecta mutants, suggesting that this tuning arises from passive mechanical properties. The radial tuning of the RL and TM contributes to the selective stimulation of OHCs, playing a role in cochlear frequency selectivity (10). The role of TM in enhancing the mechanical deflection of hair bundles is limited (11).

### 2.4 Hearing loss and TM

Abnormalities in the structure and function of the TM can contribute to hearing loss, particularly through the degeneration of TM or loss of horizontal top connectors. While changes in the TM affect its elasticity and stiffness, impairing cochlear amplification (4), it should be noted that hearing loss is not directly attributable to TM dysfunction. Current diagnostic tools, such as otoacoustic emissions (OAEs), provide indirect insights into TM function, but direct clinical assessment methods for TM integrity are lacking (12). Further research is needed to understand how TM dysfunction contributes to hearing loss and to develop better diagnostic techniques.

## 3 Common causes of TM abnormalities

### 3.1 Idiopathic sudden deafness

Several studies have reported idiopathic sudden hearing loss cases exhibit abnormal TM through temporal bone dissect (1, 13–15). Fred H Linthicum Jr. et al. suggested that the analysis of temporal bones from patients with sudden sensorineural hearing loss does not support a vascular insufficiency, but rather points to a viral etiology (14). Y Nomura et al. demonstrated that direct inoculation of herpes simplex virus (HSV) into the guinea pig scala tympani induced morphological changes in the TM, including atrophy, roll-up, and dot formation, confirmed by immunofluorescent and electron microscopic evidence of HSV infection (16). Therefore, we consider whether the TM has a virus susceptibility, and the virus destroys the rigid structure of the TM, resulting in the TM losing support, curling and other morphological abnormalities. Viral infection results in malformation of the operculum, contributing to sudden hearing loss. However, the specific pathogenesis requires further investigation.

### 3.2 Genetic etiologies

Hereditary mutations in genes encoding the TM proteins are significant contributors to TM abnormalities and associated hearing loss. These mutations impair the integrity, leading to sensorineural hearing loss, with phenotypic differences depending on the affected gene and mutation site.

#### 3.2.1 *TECTA* (*α*-tectorin): a central player in TM structure

*TECTA*, located on chromosome 11q23.3, encodes α-tectorin, a major non-collagenous protein essential for the organization and stability of collagen fibrils in the TM. Mutations in *TECTA* are associated with autosomal dominant (DFNA8/12) and autosomal recessive (DFNB21) nonsyndromic hearing loss (17, 18), as well as with Jacobsen syndrome (19). *α*-Tectorin consists of three major domains, including the entactin domain (ENT domain), zonadhesin domain (ZA domain) and zona pellucida (ZP domain). Genotype–phenotype correlations are not strictly one-to-one but show diverse patterns, with variant-specific correlations being more prominent (18). For instance, mutations in the ZA domain are linked to high-frequency hearing loss by impairing TM-hair cell interactions, which are critical for cochlear tuning. Mutations in the ZP domain result in mid-frequency hearing loss, likely due to disruption of the striated-sheet matrix essential for TM integrity. Mutations in the ENT domain is correlated with mid-frequency or high-frequency sensorineural hearing loss. Studies show that *TECTA* mutations destabilize collagen networks and reduce TM elasticity, thereby impairing sound wave propagation and amplification (20). Furthermore, experimental evidence suggests that *TECTA*, in conjunction with genes such as *MYO6*, *MYO7A*, and *PTPRQ*, may contribute to age-related hearing loss (21).

#### 3.2.2 *TECTB* (*β*-tectorin): modulating cochlear sensitivity

*TECTB*, located on chromosome 10q25.2, encoding β-tectorin, is a glycoprotein that modulates TM motion, particularly its radial displacement. AS a candidate gene for hearing loss, *TECTB* may act downstream of *Gata3* in cochlear supporting cells, with altered expression contributing to sensorineural deafness in hypoparathyroidism, sensorineural hearing loss, and renal disease (HDR) syndrome (22). Mutations in *TECTB* affect cochlear tuning and spontaneous otoacoustic emissions (SOAEs), as demonstrate in *TECTB*-null mouse models. These findings highlight the importance of *β*-tectorin in TM biomechanics and auditory function (23). Future research should focus on exploring its potential as a diagnostic marker and its involvement in broader auditory conditions.

#### 3.2.3 *CEACAM16* (CECAM16): stabilizing the TM matrix

*CEACAM16*, located on 19q13.31-q13.32, encoded CECAM16. CEACAM16 stabilizes the TM matrix through interactions with *α*-and *β*-tectorin (24). Mutations in *CEACAM16* are associated with progressive hearing loss in DFNA4B and DFNB113 (25, 26). DFNA4B shows autosomal dominant, postlingual, severe to profound, progressive sensorineural hearing loss (27). DFNB113 shows autosomal recessive, postlingual, mild to moderate progressive sensorineural hearing loss (28). Functional studies reveal that *CEACAM16* mutations lead to accelerated TM degradation and impaired hair cell activation (26). Studies have shown that CEACAM16-deficient (*CEACAM16*^−/−^) rats exhibit increased spontaneous otoacoustic emissions (SOAEs) compared to normal rats, further supporting its role in TM integrity (12, 23). *CEACAM16* mutations underlie progressive forms of sensorineural hearing loss, emphasizing the need for genetic screening and animal models to explore its therapeutic potential. Further research on its interaction with other TM proteins (*α*-and *β*-tectorin) may provide deeper insights into its function and potential interventions.

#### 3.2.4 *COL11A1*, *COL11A2* and *COL2A1* (collagen isoforms): structural integrity of TM

Collagen XI isoforms, encoded by *COL11A1*, *COL11A2*, and *COL2A1*, are critical for the radial collagen fibrils of the TM. Mutations in these genes cause syndromic hearing loss (e.g., Stickler syndrome) and nonsyndromic forms like DFNA37 (29–33). Defective collagen fibrils result in weakened TM structure and diminished auditory signal transmission (34). The effects of collagen XI mutations underscore the importance of genetic screening for both syndromic and nonsyndromic hearing loss. Insights into the molecular role of collagen XI in TM mechanics provide opportunities for targeted interventions and therapeutic advancements.

#### 3.2.5 *OTOG* and *OTOGL* (otogelin and otogelin-like): non-collagenous components of the TM

*OTOG*, located on chromosome 11p15.1, encode otogelin. Otogelin is a critical component of the acellular membranes in the inner ear. Otogelin-like, sharing 33.3% amino acid identity with otogelin, is encoded by *OTOGL* (located on chromosome 12q21.31) (35). Both proteins are non-collagenous components of the TM, cochlea, vestibule, utricular and saccular maculae, and cristae ampullaris of the semicircular canals (35, 36). Otogelin and otogelin-like are essential for maintaining the structural integrity of the TM and outer hair cell (OHC) bundles. Their absence leads to the loss of horizontal top connectors in OHC bundles and prevents their anchorage to the attachment crowns of the TM (3). Otogelin is involved in early TM development, while otogelin-like contributes to later stages of TM maturation (3). Mutations in *OTOG* cause DFNB18B, an autosomal recessive, prelingual, moderate-to-severe, mid-frequency sensorineural hearing loss (37). Mutations in *OTOGL* are linked to DFNB84B, an autosomal recessive, prelingual, moderate, mid-to high-frequency sensorineural hearing loss (38). Studies have linked *OTOG* mutations to noise-induced hearing loss and familial Meniere’s disease, while *OTOG* and *OTOGL* variants have been associated with vertigo risk (39, 40).

#### 3.2.6 *OTOA* (otoancorin): anchoring the TM to the spiral limbus

*OTOA*, located on chromosome 16p12.2, encode otoancorin. Otoancorin is a non-collagenous glycoprotein required for the adhesion of the TM to the Spiral Limbus. Otoancorin is expressed near the inner hair cell (IHC) cuticular plate and may play a role in attaching IHC stereocilia to the TM. It ensures proper anchoring and structural stability of the TM, which is critical for auditory signal transmission (6). Mutations in *OTOA* are responsible for DFNB22, an autosomal recessive, prelingual, moderate-to-profound sensorineural hearing loss (41, 42). Pathogenic mutations often convert *OTOA* to its pseudogene *OTOAP1*, resulting in transcription termination and loss of otoancorin function (43). Loss of otoancorin disrupts TM adhesion, impairing cochlear mechanics. This has been supported by studies showing premature transcription termination due to pseudogene conversion.

#### 3.2.7 *STRC* (stereocilin): critical for TM-hair cell attachments

*STRC*, located on chromosome 15q15.3, encode Stereocilin. Stereocilin is a protein localized to the stereocilia of OHCs in the inner ear, where it associates with horizontal top connectors and TM attachment crowns (44). It plays a crucial role in anchoring the tallest OHC stereocilia to the underside of theTM. Mutations in *STRC* lead to DFNB16 and Deafness-Infertility Syndrome (DIS) (45, 46). DFNB16 is characterized by autosomal recessive, mild-to-moderate, mid-to-high frequency progressive sensorineural hearing loss, typically presenting in early childhood. DIS manifests as childhood deafness in both sexes and exclusive male infertility, caused by deletion of *STRC* and *CATSPER2*. The *STRC* gene includes a pseudogene with 99.6% conserved coding sequence, complicating diagnostic implementation. Copy number variations (CNVs) in STRC and OTOA account for 73 and 13% of CNVs identified in non-syndromic hearing loss (NSHL), respectively (47). Due to gene-pseudogene conversions and the limitations of next-generation sequencing (NGS) in detecting CNVs, the carrier frequency of deleterious CNVs in *STRC* may be underestimated.

#### 3.2.8 *TUB* (TUB): regulating stereociliary integrity

*TUB*, located on chromosome 11p15.4, encode TUB. The TUB protein is essential for maintaining the localization of stereocilin at the tips of stereocilia and ensuring the integrity of stereociliary links, such as horizontal top connectors and TM attachment crowns (48). It belongs to the tubby-like protein family, characterized by a highly conserved C-terminal domain (49). TUB is associated with stereociliary link maintenance and cochlear health. Its deficiency leads to cochlear degeneration, obesity, and insulin resistance in mice, suggesting its multifaceted role in cellular function (50). Mutations in TUB are linked to syndromic conditions. For example, a Caucasian family was found to have retinal dystrophy and obesity associated with TUB mutations, indicating potential parallels between its function in humans and mice (51). While direct evidence linking TUB to hereditary hearing loss is limited, its association with TM function suggests that homozygous mutations may result in mild-to-moderate sensorineural hearing loss. Further studies are needed to confirm this speculation. Hong et al. proposed that TUB and TULP3 share similar roles in regulating cilia formation and protein trafficking. Investigating this relationship may uncover novel insights into the role of TUB in hearing loss (52).

### 3.3 Ototoxicity

Ototoxic drugs, such as aminoglycoside antibiotics (e.g., gentamicin, kanamycin), chemotherapeutic agents (e.g., carboplatin), and loop diuretics (e.g., furosemide), exert significant toxic effects on the auditory system, particularly on the structure and function of the TM in the inner ear. These drugs disrupt the transmission and amplification of auditory signals through direct or indirect effects on the TM.

#### 3.3.1 Aminoglycoside antibiotics: mechanisms of gentamicin

Gentamicin is known to cause auditory damage primarily by destroying inner ear hair cells. However, the TM also exhibits notable changes under its ototoxic effects. Studies have shown that gentamicin disrupts the fibrous connections between hair cells and the TM, preventing the hair cells from anchoring securely to the TM. This loss of connection reduces the hair cells’ responsiveness to sound wave stimuli and leads to TM detachment from the basilar membrane and localized contraction. Interestingly, this contraction may represent an adaptive repair response. In the damaged regions, the TM produces a new basal layer, which reconnects with the original TM and regenerating hair cells within 5 to 10 days following gentamicin treatment. This regenerative ability of the TM plays a critical role in partial auditory recovery. Unlike noise-induced damage, which directly destroys the TM, gentamicin primarily exerts its effects on the TM through indirect mechanisms, highlighting its unique mode of ototoxicity (53).

#### 3.3.2 Chemotherapeutic agents: carboplatin and its indirect impact

Carboplatin, a widely used chemotherapeutic agent, exhibits ototoxicity by selectively damaging IHCs, with minimal effects on OHCs. Research indicates that carboplatin indirectly affects the TM by disrupting the ionic circulation system in the cochlea. The damage to IHCs interrupts the normal flow of potassium ions (K^+^) into the endolymph, leading to dysfunction of interdental cells (IDCs). This dysfunction results in the accumulation and thickening of material in the TM’s limbal zone. Additionally, structural abnormalities in IDCs, such as collapse, dehydration, and vacuolation, further exacerbate TM dysfunction. Unlike gentamicin, which directly disrupts the fibrous connections of the TM, carboplatin’s effects rely on complex interactions among support cells, emphasizing the unique mechanisms of TM response to ototoxic injury (54, 55).

#### 3.3.3 Loop diuretics: the multifaceted impact of furosemide

Furosemide, a commonly used loop diuretic, indirectly affects cochlear structures through the inhibition of ion reabsorption in the ascending limb of the loop of Henle. Studies reveal that furosemide exposure leads to significant edema and cystic separation in the stria vascularis, resulting in a marked reduction in the endocochlear potential (EP). Additionally, furosemide induces TM collapse, which is believed to be a secondary effect of strial dysfunction. Temporal bone pathology further highlights the dose-dependent nature of furosemide-induced ototoxicity: high-dose treatments cause extensive hair cell loss and pronounced TM collapse, while low-dose treatments result in mild strial edema and cystic changes without significant TM alterations or hearing loss. These findings suggest that the effects of furosemide on the TM are multifaceted and likely mediated through disruptions in cochlear ionic circulation and mechanical coupling mechanisms (56).

The effects of ototoxic drugs on the TM involve both direct disruption of hair cell-TM connections and indirect damage through impaired cochlear support cell function and ionic circulation. Different drugs exhibit unique modes of action on the TM. For instance, gentamicin-induced regeneration of a new basal layer demonstrates the TM’s intrinsic repair capability, whereas carboplatin and furosemide aggravate TM dysfunction through complex cellular interactions and ionic imbalances. These findings underscore the critical role of the TM in ototoxic drug-induced auditory pathology and highlight its potential as a therapeutic target. Future strategies may focus on restoring ionic circulation and support cell function to mitigate ototoxic damage and improve auditory outcomes.

### 3.4 Presbycusis

As aging progresses, significant structural changes occur in the inner ear, closely associated with the development of age-related hearing loss (ARHL) or presbycusis. Studies have shown that in both aged Fischer 344 (F344) rats and human cochleae, the TM undergoes substantial degeneration. These changes include a decline in the density of the core matrix, the loss of non-collagenous glycoproteins such as *TECTA*, *TECTB*, and *CEACAM16*, and the separation of the TM from the spiral ligament. These alterations weaken the TM’s mechanical properties, such as mass and stiffness, thereby impairing its ability to amplify sound-induced motion and perform frequency selectivity. In F344 rats, the TM exhibits pronounced deformation and detachment from the organ of Corti. Similarly, in human samples, the characteristic TM degeneration highlights its critical role in the aging process (57). TM degeneration may precedes hair cell loss and elevated hearing thresholds, suggesting its potential involvement in the early stages of ARHL. Aging IHCs display phenomena such as stereocilia fusion, elongation, and internalization, which may further disrupt the mechanical transduction of sound signals. Together, these changes in the TM and IHC stereocilia exacerbate the decline in auditory function (58). These findings underscore the critical role of the TM in the progression of ARHL. However, the exact contributions of IHC stereocilia changes and their interactions with TM degeneration require further study. The importance of targeting these structures for potential therapeutic strategies to delay or reverse age-related hearing decline is clear, but additional research is necessary to fully understand these complex mechanisms and their therapeutic implications.

### 3.5 Thyroid hormone in the development of the tectorial membrane

Thyroid hormone (TH) is an indispensable regulator in cochlear development, and its deficiency leads to significant structural abnormalities in the TM and profound auditory dysfunction. Studies have demonstrated that TH regulates the normal development of the TM by modulating the transformation of Kolliker’s organ (KO). Under normal conditions, TH induces the transition of KO cells from tall columnar cells to supporting cells, facilitates the formation of the tunnel of Corti and Nuel’s spaces, and terminates the secretory activity of KO. However, in hypothyroidism, the development of KO is arrested, and its persistent secretory activity results in a markedly enlarged, morphologically distorted TM with disorganized distributions of glycoproteins and carbohydrates. These abnormalities may impair the TM’s mechanical properties, weakening its ability to amplify and transmit auditory signals (59).

TH deficiency also disrupts the composition of TM components, including an increase in *β*-tectorin levels and abnormalities in the striated-sheet matrix structure, further reducing the TM’s mechanical functionality. Moreover, chronic damage to OHCs occurs, characterized by a permanent reduction in the expression of *KCNQ4* potassium channels and delayed maturation of Prestin protein. In addition, reduced *KCNJ10* channel expression in the stria vascularis of the spiral ganglion leads to a significant decrease in endocochlear potential (EP), further compromising hair cell function. These changes culminate in irreversible auditory deficits (60).

Mutations in the *SLC26A4* gene, such as those associated with Pendred syndrome and DFNB4, profoundly impact TM structure and function through both TH-dependent and TH-independent mechanisms. In *Slc26a4*^loop/loop^ mice, although serum T3 and T4 levels remain normal, thyroid follicles exhibit marked atrophy, the TM becomes significantly thickened, *β*-tectorin expression is reduced, and cochlear bone mineralization is impaired (61). These defects are also associated with the loss of BK potassium channels in inner hair cells. Such abnormalities may result from localized TH deficiency due to inner ear fluid acidification, which impairs the activity of pH-sensitive enzymes such as type II deiodinase (D2) and reduces the conversion of T4 to T3, thereby exacerbating hearing loss.

Congenital hypothyroidism further highlights the regulatory role of TH in TM development. The TM in hypothyroid cochleae displays an abnormally enlarged and distorted morphology, with significantly disorganized distributions of glycoproteins and carbohydrates. Radiolabeled studies have revealed that the TM in hypothyroid rats exhibits a markedly increased uptake of N-acetyl-D-glucosamine, indicating dysregulated glycoprotein synthesis and secretion. These findings underscore the critical role of TH in the development of the TM, as well as in the synthesis and secretion of glycoproteins. TH deficiency may be closely associated with age-related hearing loss (presbycusis) and other auditory dysfunctions (62).

## 4 Clinical implications

### 4.1 Diagnostic strategies

Early diagnosis of hearing loss caused by TM abnormalities is crucial for timely prevention and treatment. For patients with a family history of mid-or high-frequency hearing loss, screening for genes associated with TM function may be considered. Detection of spontaneous otoacoustic emissions (SOAE) or stimulus-frequency otoacoustic emissions (SFOAE) can help identify functional changes in outer hair cells caused by TM abnormalities at an early stage. Structural or functional abnormalities in the TM may lead to either an enhancement or a loss of OAE signals, making these diagnostic methods significantly important for early detection of potential issues. In addition, optical coherence tomography (OCT) technology provides high-resolution cochlear imaging, which aids clinicians in detecting subtle changes in the TM and other crucial cochlear structures. While this technology is primarily used in research, it holds potential as an effective tool for early diagnosis of TM abnormalities. Notably, OCT has been utilized in some hospitals for the assessment of otitis media, and its application may expand to hearing diagnostics in the future.

### 4.2 Electrocochleography: a tool for indirectly evaluating tectorial membrane function

Electrocochleography (ECochG) is a clinical diagnostic tool commonly used to assess cochlear function, particularly the mechanical and electrical responses of the OHCs and IHCs. ECochG includes four distinct signal components: the cochlear microphonic (CM), which represents the response from outer hair cells; the auditory nerve neurophonic (ANN), reflecting the early neural response and activity from inner hair cells; the compound action potential (CAP), which is the early response from the auditory nerve; and the summating potential (SP), primarily associated with the response of inner hair cells (63–67). ECochG, while not directly assessing Tectorial Membrane (TM) integrity, can serve as an indirect method for evaluating TM function by reflecting the mechanical coupling between the TM and hair cells. Changes in TM function, such as altered OHC stereocilia motion or disrupted IHC activation, can affect the cochlear response, which is captured in the ECochG signals. Specifically, Cochlear Microphonic (CM) and Compound Action Potential (CAP) reflect the OHC response, while the Summating Potential (SP) primarily reflects IHC activity. Alterations in TM function, including disrupted mechanotransduction or calcium regulation, can lead to changes in these signals. ECochG is therefore a useful tool for indirectly assessing TM-related dysfunction by detecting deviations in these cochlear potentials, which can provide insights into the TM’s impact on cochlear amplification and mechanotransduction.

### 4.3 Therapeutic approaches

Currently, the primary treatment options for hearing loss caused by TM damage are still focused on assistive hearing devices, such as hearing aids and cochlear implants. In addition to these traditional methods, gene therapy has emerged as a new area of research, particularly the development of treatments targeting deafness-related genes associated with the TM. Currently, gene therapies mainly include gene editing techniques and gene replacement therapy. At the same time, some researchers are exploring the potential of small molecule drugs for treatment. For patients with TM damage caused by immune responses or inflammation, anti-inflammatory drugs may be used to reduce inflammation and prevent further degeneration. Additionally, stem cell therapy has shown potential application value in cochlear injury repair.

### 4.4 Future directions

Future research should focus on optimizing the diagnosis and treatment of hearing loss associated with the TM. In the field of gene therapy, studies on genes such as *TECTA*, *TECTB*, and *CEACAM16* could advance the development of CRISPR-Cas9, RNA interference (RNAi), and gene replacement therapies. Pharmacological interventions may explore small molecules to stabilize the TM structure or employ anti-inflammatory and immune modulation to prevent inflammatory damage. Furthermore, stem cell therapy and tissue engineering could provide new approaches for TM regeneration. Advanced imaging technologies, such as optical coherence tomography (OCT), combined with artificial intelligence, can enhance early diagnostic accuracy, while ion homeostasis regulation may help restore the mechanical properties and auditory function of the TM. Ultimately, precision medicine, integrating genomics and machine learning, will drive the development of personalized treatment plans, making the treatment of TM-related hearing loss more efficient and precise.

## 5 Conclusion

The TM plays a critical role in the auditory system, and its structural and functional abnormalities are closely associated with various forms of hearing loss. Although significant progress has been made in recent years regarding TM research, particularly in terms of gene mutations, molecular mechanisms, and biomechanical properties, many mysteries remain unresolved. This review discusses the composition, function, related disease mechanisms, and potential therapeutic strategies for TM, as well as future research directions.

Currently, gene therapy, stem cell therapy, small molecule drugs, and tissue engineering offer new possibilities for treating TM damage, although these methods are still in the research phase and lack mature clinical applications. Meanwhile, advanced imaging technologies, such as optical coherence tomography (OCT), combined with artificial intelligence-assisted analysis, hold promise for improving the early diagnostic capability of TM abnormalities and laying the foundation for precision medicine. However, further exploration is needed to better understand the biomechanical properties of TM, its genetic regulation mechanisms, and its interactions with external environmental factors in order to develop more effective interventions.

In the future, interdisciplinary collaboration (including molecular biology, genetics, acoustic engineering, and clinical medicine) will be key to advancing TM research and hearing loss treatment. With the development of personalized treatment, gene editing, and regenerative medicine, the treatment of TM-related hearing disorders is expected to become more precise and efficient, offering improved auditory restoration for patients.

## References

[1] Ishai R Kamakura T Nadol JB Jr. Abnormal tectorial membranes in sensorineural hearing loss: a human temporal bone study. Otol Neurotol. (2019) 40:e732–8. doi: 10.1097/mao.0000000000002286, PMID: 31219968

[2] Gavara N Manoussaki D Chadwick RS. Auditory mechanics of the tectorial membrane and the cochlear spiral. Curr Opin Otolaryngol Head Neck Surg. (2011) 19:382–7. doi: 10.1097/MOO.0b013e32834a5bc9, PMID: 21785353 PMC3327783

[3] Avan P Gal SL Michel V Dupont T Hardelin J-P Petit C. Otogelin, otogelin-like, and stereocilin form links connecting outer hair cell stereocilia to each other and the tectorial membrane. Proc Natl Acad Sci USA. (2019) 116:25948–57. doi: 10.1073/pnas.1902781116, PMID: 31776257 PMC6926040

[4] Cartagena-Rivera AX Le Gal S Richards K Verpy E Chadwick RS. Cochlear outer hair cell horizontal top connectors mediate mature stereocilia bundle mechanics. Sci Adv. (2019) 5:eaat9934. doi: 10.1126/sciadv.aat9934, PMID: 30801007 PMC6382404

[5] Guinan JJ Jr. How are inner hair cells stimulated? Evidence for multiple mechanical drives. Hear Res. (2012) 292:35–50. doi: 10.1016/j.heares.2012.08.005, PMID: 22959529 PMC3549570

[6] Hakizimana P Fridberger A. Inner hair cell stereocilia are embedded in the tectorial membrane. Nat Commun. (2021) 12:2604. doi: 10.1038/s41467-021-22870-1, PMID: 33972539 PMC8110531

[7] Ghaffari R Aranyosi AJ Freeman DM. Longitudinally propagating traveling waves of the mammalian tectorial membrane. Proc Natl Acad Sci USA. (2007) 104:16510–5. doi: 10.1073/pnas.0703665104, PMID: 17925447 PMC2034249

[8] Ghaffari R Aranyosi AJ Richardson GP Freeman DM. Tectorial membrane travelling waves underlie abnormal hearing in Tectb mutant mice. Nat Commun. (2010) 1:96. doi: 10.1038/ncomms1094, PMID: 20981024 PMC2982163

[9] Lim DJ. Cochlear anatomy related to cochlear micromechanics. A review *J Acoust Soc Am*. (1980) 67:1686–95. doi: 10.1121/1.384295, PMID: 6768784

[10] Lee HY Raphael PD Xia A Kim J Grillet N Applegate BE. Two-dimensional Cochlear micromechanics measured in vivo demonstrate radial tuning within the mouse organ of Corti. J Neurosci. (2016) 36:8160–73. doi: 10.1523/jneurosci.1157-16.2016, PMID: 27488636 PMC4971363

[11] Meenderink SWF Lin X Park BH Dong W. Sound induced vibrations deform the organ of Corti complex in the low-frequency apical region of the gerbil cochlea for Normal hearing: sound induced vibrations deform the organ of Corti complex. J Assoc Res Otolaryngol. (2022) 23:579–91. doi: 10.1007/s10162-022-00856-0, PMID: 35798901 PMC9613840

[12] Cheatham MA Ahmad A Zhou Y Goodyear RJ Dallos P Richardson GP. Increased spontaneous Otoacoustic emissions in mice with a detached tectorial membrane. J Assoc Res Otolaryngol. (2016) 17:81–8. doi: 10.1007/s10162-015-0551-7, PMID: 26691158 PMC4791414

[13] Sando I Loehr A Harada T Sobel JH. Sudden deafness: histopathologic correlation in temporal bone. Ann Otol Rhinol Laryngol. (1977) 86:269–79. doi: 10.1177/000348947708600301, PMID: 869429

[14] Linthicum FH Jr Doherty J Berliner KI. Idiopathic sudden sensorineural hearing loss: vascular or viral? Otolaryngol Head Neck Surg. (2013) 149:914–7. doi: 10.1177/0194599813506546, PMID: 24067949 PMC4068115

[15] Inagaki T Cureoglu S Morita N Terao K Sato T Suzuki M. Vestibular system changes in sudden deafness with and without vertigo: a human temporal bone study. Otol Neurotol. (2012) 33:1151–5. doi: 10.1097/MAO.0b013e3182635440, PMID: 22872175 PMC3874473

[16] Nomura Y Kurata T Saito K. Cochlear changes after herpes simplex virus infection. Acta Otolaryngol. (1985) 99:419–27. doi: 10.3109/00016488509108933, PMID: 2990152

[17] Yasukawa R Moteki H Nishio SY Ishikawa K Abe S Honkura Y. The prevalence and clinical characteristics of TECTA-associated autosomal dominant hearing loss. Genes. (2019) 10:744. doi: 10.3390/genes10100744, PMID: 31554319 PMC6826443

[18] Choi BY Kim J Chung J Kim AR Mun SJ Kang SI. Whole-exome sequencing identifies a novel genotype-phenotype correlation in the entactin domain of the known deafness gene TECTA. Plo S one. (2014) 9:e97040. doi: 10.1371/journal.pone.0097040, PMID: 24816743 PMC4016231

[19] Fryns JP Kleczkowska A Buttiens M Marien P van den Berghe H. Distal 11q monosomy. The typical 11q monosomy syndrome is due to deletion of subband 11q24.1. Clin Genet. (1986) 30:255–60. doi: 10.1111/j.1399-0004.1986.tb00605.x, PMID: 3791674

[20] Kim DK Kim JA Park J Niazi A Almishaal A Park S. The release of surface-anchored α-tectorin, an apical extracellular matrix protein, mediates tectorial membrane organization. Sci Adv. (2019) 5:eaay6300. doi: 10.1126/sciadv.aay6300, PMID: 31807709 PMC6881170

[21] Boucher S Tai FWJ Delmaghani S Lelli A Singh-Estivalet A Dupont T. Ultrarare heterozygous pathogenic variants of genes causing dominant forms of early-onset deafness underlie severe presbycusis. Proc Natl Acad Sci USA. (2020) 117:31278–89. doi: 10.1073/pnas.2010782117, PMID: 33229591 PMC7733833

[22] Xu J Yu D Dong X Xie X Xu M Guo L. GATA3 maintains the quiescent state of cochlear supporting cells by regulating p 27 (kip 1). Sci Rep. (2021) 11:15779. doi: 10.1038/s41598-021-95427-3, PMID: 34349220 PMC8338922

[23] Cheatham MA. Spontaneous otoacoustic emissions are biomarkers for mice with tectorial membrane defects. Hear Res. (2021) 409:108314. doi: 10.1016/j.heares.2021.108314, PMID: 34332206 PMC8419146

[24] Cheatham MA Goodyear RJ Homma K Legan PK Korchagina J Naskar S. Loss of the tectorial membrane protein CEACAM16 enhances spontaneous, stimulus-frequency, and transiently evoked otoacoustic emissions. J Neurosci. (2014) 34:10325–38. doi: 10.1523/jneurosci.1256-14.2014, PMID: 25080593 PMC4115139

[25] Markova TG Alekseeva NN Ryzhkova OP Shatokhina OL Orlova AA Zabnenkova VV. Auditory phenotype of a novel missense variant in the CEACAM16 gene in a large Russian family with autosomal dominant nonsyndromic hearing loss. J Int Adv Otol. (2024) 20:119–26. doi: 10.5152/iao.2024.231252, PMID: 39157884 PMC11114206

[26] Goodyear RJ Cheatham MA Naskar S Zhou Y Osgood RT Zheng J. Accelerated age-related degradation of the tectorial membrane in the Ceacam 16 (βgal/βgal) null mutant mouse, a model for late-onset human hereditary deafness DFNB113. Front Mol Neurosci. (2019) 12:147. doi: 10.3389/fnmol.2019.00147, PMID: 31249509 PMC6582249

[27] Wang H Wang X He C Li H Qing J Grati M’. Exome sequencing identifies a novel CEACAM16 mutation associated with autosomal dominant nonsyndromic hearing loss DFNA4B in a Chinese family. J Hum Genet. (2015) 60:119–26. doi: 10.1038/jhg.2014.114, PMID: 25589040 PMC4375019

[28] Booth KT Kahrizi K Najmabadi H Azaiez H Smith RJ. Old gene, new phenotype: splice-altering variants in *CEACAM16* cause recessive non-syndromic hearing impairment. J Med Genet. (2018) 55:555–60. doi: 10.1136/jmedgenet-2018-105349, PMID: 29703829 PMC6060001

[29] Booth KT Askew JW Talebizadeh Z Huygen PLM Eudy J Kenyon J. Splice-altering variant in COL11A1 as a cause of nonsyndromic hearing loss DFNA37. Genet Med. (2019) 21:948–54. doi: 10.1038/s41436-018-0285-0, PMID: 30245514 PMC6431578

[30] Ala-Kokko L Shanske AL. Mosaicism in Marshall syndrome. Am J Med Genet A. (2009) 149A:1327–30. doi: 10.1002/ajmg.a.32873, PMID: 19449424

[31] Richards AJ Yates JR Williams R Payne SJ Pope FM Scott JD. A family with stickler syndrome type 2 has a mutation in the COL11A1 gene resulting in the substitution of glycine 97 by valine in alpha 1 (XI) collagen. Hum Mol Genet. (1996) 5:1339–43. doi: 10.1093/hmg/5.9.1339, PMID: 8872475

[32] Tompson SW Bacino CA Safina NP Bober MB Proud VK Funari T. Fibrochondrogenesis results from mutations in the COL11A1 type XI collagen gene. Am J Hum Genet. (2010) 87:708–12. doi: 10.1016/j.ajhg.2010.10.009, PMID: 21035103 PMC2978944

[33] Khalifa O Imtiaz F Allam R al-Hassnan Z al-Hemidan A al-Mane K. A recessive form of Marshall syndrome is caused by a mutation in the COL11A1 gene. J Med Genet. (2012) 49:246–8. doi: 10.1136/jmedgenet-2012-100783, PMID: 22499343

[34] Sellon JB Ghaffari R Freeman DM. The tectorial membrane: mechanical properties and functions. Cold Spring Harb Perspect Med. (2019) 9:a038950. doi: 10.1101/cshperspect.a038950, PMID: 30348837 PMC6771364

[35] Yariz KO Duman D Zazo Seco C Dallman J Huang M Peters TA. Mutations in OTOGL, encoding the inner ear protein otogelin-like, cause moderate sensorineural hearing loss. Am J Hum Genet. (2012) 91:872–82. doi: 10.1016/j.ajhg.2012.09.011, PMID: 23122586 PMC3487139

[36] Simmler M-C Cohen-Salmon M El-Amraoui A Guillaud L Benichou J-C Petit C. Targeted disruption of otog results in deafness and severe imbalance. Nat Genet. (2000) 24:139–43. doi: 10.1038/72793, PMID: 10655058

[37] Schraders M Ruiz-Palmero L Kalay E Oostrik J del Castillo FJ Sezgin O. Mutations of the gene encoding otogelin are a cause of autosomal-recessive nonsyndromic moderate hearing impairment. Am J Hum Genet. (2012) 91:883–9. doi: 10.1016/j.ajhg.2012.09.012, PMID: 23122587 PMC3487128

[38] Zhang X Ni Y Liu Y Zhang L Zhang M Fang X. Screening of noise-induced hearing loss (NIHL)-associated SNPs and the assessment of its genetic susceptibility. Environ Health. (2019) 18:30. doi: 10.1186/s12940-019-0471-9, PMID: 30947719 PMC6449917

[39] Skuladottir AT Bjornsdottir G Nawaz MS Petersen H Rognvaldsson S Moore KHS. A genome-wide meta-analysis uncovers six sequence variants conferring risk of vertigo. Communications Biology. (2021) 4:1148. doi: 10.1038/s42003-021-02673-2, PMID: 34620984 PMC8497462

[40] Roman-Naranjo P Gallego-Martinez A Soto-Varela A Aran I Moleon MC Espinosa-Sanchez JM. Burden of rare variants in the OTOG gene in familial Meniere’s disease. Ear Hear. (2020) 41:1598–605. doi: 10.1097/aud.0000000000000878, PMID: 33136635

[41] Lee K Chiu I Santos-Cortez RL Basit S Khan S Azeem Z. Novel OTOA mutations cause autosomal recessive non-syndromic hearing impairment in Pakistani families. Clin Genet. (2013) 84:294–6. doi: 10.1111/cge.12047, PMID: 23173898 PMC6220893

[42] Zwaenepoel I Mustapha M Leibovici M Verpy E Goodyear R Liu XZ. Otoancorin, an inner ear protein restricted to the interface between the apical surface of sensory epithelia and their overlying acellular gels, is defective in autosomal recessive deafness DFNB22. Proc Natl Acad Sci USA. (2002) 99:6240–5. doi: 10.1073/pnas.082515999, PMID: 11972037 PMC122933

[43] Laurent S Gehrig C Nouspikel T Amr SS Oza A Murphy E. Molecular characterization of pathogenic OTOA gene conversions in hearing loss patients. Hum Mutat. (2021) 42:373–7. doi: 10.1002/humu.24167, PMID: 33492714 PMC8750238

[44] Verpy E Leibovici M Michalski N Goodyear RJ Houdon C Weil D. Stereocilin connects outer hair cell stereocilia to one another and to the tectorial membrane. J Comp Neurol. (2011) 519:194–210. doi: 10.1002/cne.22509, PMID: 21165971 PMC3375590

[45] Verpy E Masmoudi S Zwaenepoel I Leibovici M Hutchin TP del Castillo I. Mutations in a new gene encoding a protein of the hair bundle cause non-syndromic deafness at the DFNB16 locus. Nat Genet. (2001) 29:345–9. doi: 10.1038/ng726, PMID: 11687802

[46] Vona B Hofrichter MA Neuner C Schröder J Gehrig A Hennermann JB. DFNB16 is a frequent cause of congenital hearing impairment: implementation of STRC mutation analysis in routine diagnostics. Clin Genet. (2015) 87:49–55. doi: 10.1111/cge.12332, PMID: 26011646 PMC4302246

[47] Shearer AE Kolbe DL Azaiez H Sloan CM Frees KL Weaver AE. Copy number variants are a common cause of non-syndromic hearing loss. Genome Med. (2014) 6:37. doi: 10.1186/gm554, PMID: 24963352 PMC4067994

[48] Han W Shin JO Ma JH Min H Jung J Lee J. Distinct roles of stereociliary links in the nonlinear sound processing and noise resistance of cochlear outer hair cells. Proc Natl Acad Sci USA. (2020) 117:11109–17. doi: 10.1073/pnas.1920229117, PMID: 32358189 PMC7245111

[49] Carroll K Gomez C Shapiro L. Tubby proteins: the plot thickens. Nat Rev Mol Cell Biol. (2004) 5:55–64. doi: 10.1038/nrm1278, PMID: 14708010

[50] Noben-Trauth K Naggert JK North MA Nishina PM. A candidate gene for the mouse mutation tubby. Nature. (1996) 380:534–8. doi: 10.1038/380534a0, PMID: 8606774

[51] Borman AD Pearce LR Mackay DS Nagel-Wolfrum K Davidson AE Henderson R. A homozygous mutation in the TUB gene associated with retinal dystrophy and obesity. Hum Mutat. (2014) 35:289–93. doi: 10.1002/humu.22482, PMID: 24375934 PMC4284018

[52] Hong JJ Kim KE Park SY Bok J Seo JT Moon SJ. Differential roles of tubby family proteins in ciliary formation and trafficking. Mol Cells. (2021) 44:591–601. doi: 10.14348/molcells.2021.0082, PMID: 34462398 PMC8424140

[53] Epstein JE Cotanche DA. Secretion of a new basal layer of tectorial membrane following gentamicin-induced hair cell loss. Hear Res. (1995) 90:31–43. doi: 10.1016/0378-5955(95)00141-9, PMID: 8975003

[54] Spicer SS Salvi RJ Schulte BA. Ultrastructural changes in the spiral limbus associated with carboplatin-induced ablation of inner hair cells. Cell Tissue Res. (2000) 302:1–10. doi: 10.1007/s004410000253, PMID: 11079710

[55] Spicer SS Salvi RJ Schulte BA. Ablation of inner hair cells by carboplatin alters cells in the medial K(+) flow route and disrupts tectorial membrane. Hear Res. (1999) 136:139–50. doi: 10.1016/s0378-5955(99)00118-5, PMID: 10511633

[56] Santos F Nadol JB. Temporal bone histopathology of furosemide ototoxicity. Laryngoscope Investig Otolaryngol. (2017) 2:204–7. doi: 10.1002/lio2.108, PMID: 29085910 PMC5655552

[57] Buckiova D Popelar J Syka J. Collagen changes in the cochlea of aged Fischer 344 rats. Exp Gerontol. (2006) 41:296–302. doi: 10.1016/j.exger.2005.11.010, PMID: 16427232

[58] Bullen A Forge A Wright A Richardson GP Goodyear RJ Taylor R. Ultrastructural defects in stereocilia and tectorial membrane in aging mouse and human cochleae. J Neurosci Res. (2020) 98:1745–63. doi: 10.1002/jnr.24556 31762086

[59] Legrand C Bréhier A Clavel MC Thomasset M Rabié A. Cholecalcin (28-kDa CaBP) in the rat cochlea. Development in normal and hypothyroid animals. An immunocytochemical study. Brain Res. (1988) 38:121–9. doi: 10.1016/0165-3806(88)90090-9, PMID: 3342324

[60] Mustapha M Fang Q Gong TW Dolan DF Raphael Y Camper SA. Deafness and permanently reduced potassium channel gene expression and function in hypothyroid pit 1dw mutants. J Neurosci. (2009) 29:1212–23. doi: 10.1523/jneurosci.4957-08.2009 19176829 PMC3862029

[61] Dror AA Lenz DR Shivatzki S Cohen K Ashur-Fabian O Avraham KB. Atrophic thyroid follicles and inner ear defects reminiscent of cochlear hypothyroidism in Slc 26a4-related deafness. Mamm Genome. (2014) 25:304–16. doi: 10.1007/s00335-014-9515-1, PMID: 24760582 PMC5944359

[62] Remezal M Gil-Loyzaga P. Incorporation of D3H glucosamine to the adult and developing cochlear tectorial membrane of normal and hypothyroid rats. Hear Res. (1993) 66:23–30. doi: 10.1016/0378-5955(93)90256-z, PMID: 8473243

[63] Davis H Deatherage BH Eldredge DH Smith CA. Summating potentials of the cochlea. Am J Phys. (1958) 195:251–61. doi: 10.1152/ajplegacy.1958.195.2.251, PMID: 13583157

[64] Zheng XY Ding DL McFadden SL Henderson D. Evidence that inner hair cells are the major source of cochlear summating potentials. Hear Res. (1997) 113:76–88. doi: 10.1016/s0378-5955(97)00127-5, PMID: 9387987

[65] Snyder RL Schreiner CE. The auditory neurophonic: basic properties. Hear Res. (1984) 15:261–80. doi: 10.1016/0378-5955(84)90033-9 6501114

[66] Chertoff M Lichtenhan J Willis M. Click-and chirp-evoked human compound action potentials. J Acoust Soc Am. (2010) 127:2992–6. doi: 10.1121/1.3372756, PMID: 21117748 PMC3188627

[67] Scott WC Giardina CK Pappa AK Fontenot TE Anderson ML Dillon MT. The compound action potential in subjects receiving a Cochlear implant. Otol Neurotol. (2016) 37:1654–61. doi: 10.1097/mao.0000000000001224, PMID: 27749750 PMC5242224

